# Systematic review and network meta-analysis of pre-emptive embolization of the aneurysm sac side branches and aneurysm sac coil embolization to improve the outcomes of endovascular aneurysm repair

**DOI:** 10.3389/fcvm.2022.947809

**Published:** 2022-07-22

**Authors:** Ye Wu, Jianhan Yin, Zhang Hongpeng, Guo Wei

**Affiliations:** ^1^Department of Vascular Surgery, First Medical Center, Chinese PLA General Hospital, Beijing, China; ^2^Medical College of Chinese PLA, Beijing, China; ^3^Nankai University, Tianjin, China

**Keywords:** prophylactic embolization, inferior mesenteric artery, type II endoleak, meta-analysis, abdominal aortic aneurysm

## Abstract

**Objective:**

Previous reports have revealed a high incidence of type II endoleak (T2EL) after endovascular aneurysm repair (EVAR). The incidence of T2EL after EVAR is reduced by pre-emptive embolization of aneurysm sac side branches (ASSB) and aneurysm sac coil embolization (ASCE). This study aimed to investigate whether different preventive interventions for T2EL were correlated with suppression of aneurysm sac expansion and reduction of the re-intervention rate.

**Methods:**

The PubMed, Web of Science, MEDLINE and Embase databases, and conference proceedings were searched to identify articles on EVAR with or without embolization. The study was developed in line with the Participants, Interventions, Comparisons, Outcomes, and Study design principles and was conducted and reported in accordance with the Preferred Reporting Items for Systematic reviews and Meta-Analyses guidelines. We used network meta-analysis based on multivariate random-effects meta-analysis to indirectly compare outcomes of different strategies for embolization during EVAR.

**Results:**

A total of 31 studies met all inclusion criteria and were included in the qualitative and quantitative syntheses. The included studies were published between 2001 and 2022 and analyzed a total of 18,542 patients, including 1,882 patients who received prophylactic embolization treatment during EVAR (experimental group) and 16,660 who did not receive prophylactic embolization during EVAR (control group). The effect of pre-emptive embolization of the inferior mesenteric artery (IMA) (IMA-ASSB) in preventing T2EL was similar (relative risk [RR] 1.01, 95% confidence interval [CI] 0.38–2.63) to the effects of non-selective embolization of ASSB (NS-ASSB) and ASCE (RR 0.88, 95% CI 0.40–1.96). IMA-ASSB showed a better clinical effect in suppressing the aneurysm sac expansion (RR 0.27, 95% CI 0.09–2.25 compared with NS-ASSB; RR 0.93, 95% CI 0.16–5.56 compared with ASCE) and reducing the re-intervention rate (RR 0.34, 95% CI 0.08–1.53 compared with NS-ASSB; RR 0.66, 95% CI 0.19–2.22 compared with ASCE). All prophylactic embolization strategies improved the clinical outcomes of EVAR.

**Conclusion:**

Prophylactic embolization during EVAR effectively prevents T2EL, suppresses the aneurysm sac expansion, and reduces the re-intervention rate. IMA embolization demonstrated benefits in achieving long-term aneurysm sac stability and lowering the risk of secondary surgery. NS-ASSB more effectively reduces the incidence of T2EL, while IMA embolization alone or in combination with ASCE enhances the clinical benefits of EVAR. In addition, as network meta-analysis is still an indirect method based on a refinement of existing data, more studies and evidence are still needed in the future to establish more credible conclusions.

## Introduction

Endovascular aneurysm repair (EVAR) has become the accepted standard therapy for abdominal aortic aneurysm (AAA) because of its low perioperative mortality and minimal invasiveness (1). However, the long-term outcomes of EVAR have not been fully elucidated and remain controversial. Recent clinical trials have shown that EVAR no longer achieves an early survival benefit compared with open surgery, because of a significant increase in death from secondary aneurysm rupture mostly caused by endoleaks (2) and a higher rate of secondary intervention (3).

Among several types of endoleaks, the most common is type II endoleak (T2EL) (4, 5). T2EL occurs because of retrograde perfusion of the AAA sac from the inferior mesenteric artery (IMA), lumbar arteries (LAs), middle sacral artery, or aberrant renal arteries (6). Most T2ELs resolve spontaneously with time and it remains debatable whether T2ELs requires aggressive therapy that may be associated with adverse late outcomes (7–11). A previous study demonstrated that 9.8% of aortic ruptures after EVAR were caused by T2EL (12), while a meta-analysis of 32 studies and 21,744 patients showed that 0.9% of patients with an isolated T2EL had a ruptured AAA (13). A reduction in the incidence of T2EL may improve the prognosis and decrease the psychological and economic burden of patients undergoing EVAR (14).

The role of pre-emptive embolization of aneurysm sac side branches (ASSB) in preventing T2EL has been debated for the past two decades. Early evidence in 2001 suggested that additional ASSB is unnecessary because of the uncertain incidence of T2EL during follow-up (15). However, recent progress in preoperative optimization, medical devices, and high-quality supportive care has led to significantly improved clinical outcomes of ASSB. Despite the lack of high-quality evidence, the latest meta-analysis suggests that ASSB aids in preventing T2EL, aneurysm sac enlargement, and re-intervention (16). The other prophylactic embolization treatment implemented during EVAR is aneurysm sac coil embolization (ASCE). To date, there is still no generalized and commonly accepted standard method of ASCE (11). However, studies have shown that ASCE effectively prevents T2EL, particularly during the mid-term follow-up of high-risk patients (17–20). A meta-analysis of 17 studies with 2,084 participants also demonstrated the safety and effectiveness of ASCE in preventing T2EL (21).

The most common sources of T2EL are the IMA and LAs. However, there is still a lack of clinical trials directly comparing the efficacy of non-selective embolization of patent aortic side branches versus embolization of the IMA alone. Furthermore, few studies have directly compared ASSB and ASCE. There was no recording and follow-up of other arteries such as accessory renal arteries in the studies that we consulted and incorporated. In the present study, we aimed to perform a network meta-analysis to compare the efficacy and basic outcomes of different prophylactic embolization treatments in IMA, Las, and sac embolization during EVAR.

## Methods

### Study design

The study was developed in line with the Participants, Interventions, Comparisons, Outcomes, Study design principles and was conducted and reported in accordance with the Preferred Reporting Items for Systematic reviews and Meta-Analyses guidelines (22).

### Participants

The participants were patients of any age with AAA who underwent EVAR with or without prophylactic embolization comprising either ASSB or ASCE. ASSB was divided into preoperative pre-emptive embolization of the IMA (IMA-ASSB) and non-selective preoperative embolization of the IMA and LAs (NS-ASSB). Few studies reported embolization of the median sacral artery or accessory renal artery.

### Interventions

EVAR with IMA-ASSB, NS-ASSB, or ASCE.

### Comparison intervention

EVAR without prophylactic embolization treatment.

### Outcomes

#### Primary outcomes

a. Incidence of T2EL during follow-up. The presence of T2EL was defined as the temporary or permanent appearance of T2EL during postoperative follow-up examination.

b. Incidence of enlargement of the diameter or size of the aneurysm sac during follow-up.

#### Secondary outcomes

a. Late all-cause-related mortality.

b. Rate of re-intervention during follow-up.

One thing to note, only a few studies mentioned their observation and follow-up of complications which was not sufficient for statistical analysis.

### Eligibility criteria

1. Inclusion criteria: retrospective or prospective studies evaluating the effect of ASSB or ASCE during EVAR compared with a control group or not that underwent no preventive intervention measures and had the patent collateral arteries retained; no date or limit on patients or publications; follow-up period of not less than 6 months; outcome indicators included the occurrence of T2EL diagnosed by contrast-enhanced CT, CTA, MRA, or other appropriate imaging examination.

2. Clinical exclusion criteria: flawed study design, implementation process, or statistical methods; funding organization influenced the study design or the implementation, analysis, or interpretation of data; incomplete data in original articles that could not be refined; case report or studies about embolization of other arteries, such as the median sacral artery or accessory renal artery which without intervention to IMA or LAs; prophylactic embolization in animals; fewer than 10 patients per group; Embolization performed in second operations.

3. Non-clinical exclusion criteria: overlapping series (only the latest publication of serial reports of a certain cohort was included); non-original article (i.e., review, case report, editorial).

### Information sources

Multiple electronic health databases (MEDLINE, Embase, PubMed, and Web of Science) were searched to identify relevant articles published from 1 October 2001 to 11 May 2022.

### Search strategy

The MEDLINE, Embase, PubMed, and Web of Science databases were searched with an unrestricted search strategy that applied a combination of Medical Subject Headings and keywords combined with the Boolean operators AND or OR to retrieve relevant reports. The following terms were used: [“aortic aneurysm abdominal” (Title/Abstract) OR “abdominal aortic aneurysms” (Title/Abstract) OR “aneurysms abdominal aortic” (Title/Abstract) OR “aortic aneurysms abdominal” (Title/Abstract) OR “abdominal aortic aneurysm” (Title/Abstract) OR “aneurysm abdominal aortic” (Title/Abstract)] AND [“embolization” (Title/Abstract) OR “embolism” (Title/Abstract) OR “embolizations” (Title/Abstract)]. Controlled trials comparing prophylactic embolization with non-intervention during EVAR were eligible for inclusion in the general meta-analysis. Single-arm studies were also retrieved for inclusion in the network meta-analysis. We adapted the terms to meet the specific requirements of the explicit search strategy used for each database. A total of 100 citations were obtained from the databases, of which 67 were excluded after browsing the titles and abstracts, yielding 33 articles for detailed review. After reviewing the full texts of the remaining studies and their cited references to identify additional studies, 32 studies were finally selected.

### Study selection

Eligibility assessment was performed independently in an unblinded standardized manner by two reviewers (Guo and Zhang).

### Data extraction and assessment of study quality

Data were extracted from the primary studies and consolidated into single spreadsheets. One author collected the data from the included articles, while another author explicitly checked the presented information. The data analyzed in the present review were all published in the included studies in case of record form or as alphanumeric text. Information filtering from relevant studies was performed manually. Study quality and risk of bias were assessed using the Newcastle Ottawa Scale (NOS) (23) (selection, comparability, and outcome) and the Cochrane Handbook for Systematic Reviews of Interventions (random sequence generation, allocation concealment, blinding of participants and personnel, blinding of outcome assessment, incomplete outcome data, selective reporting, and other bias). Two reviewers (Wu and Yin) determined the eligibility and compared the quality of the included studies. A cumulative NOS of 7 or more was considered to indicate high quality.

### Extracted data

1. Publication details: first author and year.

2. Study details: number of participating institutions, number of participants, controls and interventions, mean follow-up period, inclusion criteria, exclusion criteria, and country.

3. Participants: number of patients undergoing EVAR with or without prophylactic embolization.

4. Primary outcomes: incidences of T2EL and enlargement of the aneurysm sac during follow-up.

5. Secondary outcomes: late all-cause-related mortality and the rate of re-intervention during follow-up.

### Statistical analysis

Statistical analysis was performed in accordance with the Cochrane Handbook for Systematic Reviews, using Stata^®^ Version 16.0, RStudio (version 1.4.1103 for Windows, Boston, MA, United States), Stata package base, metan, mvmeta, metareg, network, R (version 4.1.2, R Foundation for Statistical Computing, Vienna, Austria) and R packages base, ggplot2, metafor, meta and gemtc (24). The gemtc package was used in connection with the Just Another Gibbs Sampler package to generate simulations. Statistical applications were set up to statistically analyze which prophylactic embolization treatment had the greatest effect on the progression of AAA after EVAR. There was no significant methodological heterogeneity between the datasets regarding the study design and risk of bias. The primary and secondary outcomes were analyzed as dichotomous variables by estimating the relative risk (RR) with a 95% confidence interval (CI). The I^2^ value was calculated to assess the statistical heterogeneity between studies. The heterogeneity among studies was considered significant when I^2^ was > 50%, and was considered highly significant when I^2^ was > 75%. Publication bias was assessed through funnel plots and Egger tests. The fixed-effect model based on the Mantel-Haenszel estimator was used when there was no or only slight heterogeneity among the studies. When the heterogeneity was significant, the analysis was performed with the random-effects model based on the DerSimonian-Laird estimator. The results were summarized using forest plots.

### Flow diagram of studies included in the systematic review (please refer to the Supplementary Appendix)

#### Description of studies

The search strategy retrieved a total of 3,345 articles, of which 3,314 were excluded after the title and abstract screening because they were not relevant or because they were comments only. After full-text review and data abstraction, 31 studies met all inclusion criteria and were included in the qualitative and quantitative syntheses (2, 17–20, 25–50).

One study was a prospective randomized controlled trial, while 30 were retrospective studies. All studies were published between 2001 and 2022. A total of 18,542 patients were involved in the included studies, of which 1,882 received prophylactic embolization treatment during EVAR and 16,660 did not. One study based on data from the Vascular Quality Initiative database included 15,060 patients (43). The study characteristics, study quality, and literature quality evaluation based on the NOS are summarized in Table 1. The mean NOS was 7.38 (out of a possible 9 points), suggesting that the included studies were high-quality studies. A lack of representativeness and comparability was the main reason for low NOS scores.

**TABLE 1:** General characteristics of included studies.

Author (publication year)	Study period	Nation	Study design	Embolized arteries (experimental group)	Devices of embolization	Follow up period (mean, months)	Number of patients in emboliation group	Number of patients in control group	Total number of patients in endpoint	NOS	Technical success	Re-interventions	Enlargement of sac
Parry et al. (25)	09/1996 to 04/2001	United Kingdom	Single-center Retrospective	IMA and LA	Coils	E = 24.0 C = 30.0	14	22	36	7	73.7% (14/19)	E = 0 C = 6	E = 0 C = 3
Gould et al. (15)	N.S.	United Kingdom	Single-center Retrospective	IMA and LA	Coils	E = 18.4 C = 18.9	20	43	63	6	84% (21/25)	E = 0 C = 3	E = 0 C = 4
Fabre et al. (20)	01/2009 to 12/2013	France	Single-center Retrospective	IMA and LA	Coils	24.0	83	104	187	8	100% (83/83)	N.S.	N.S.
Burbelko et al. (32)	01/2008 to 04/2010	Germany	Single-center Retrospective	IMA and LA	AVP	E = 30.1 C = 30.2	33	38	71	6	100% (33/33)	E = 0 C = 4	E = 0 C = 5
Aoki et al. (44)	10/2009 to 08/2019	Japan	Single-center Retrospective	IMA and LA	Coils	E = 53.5 C = 53.0	48	34	82	6	IMA: 96.4% (54/56) and LAs: 74.5% (108/145)	N.S.	N.S.
Chikazawa et al. (29)	05/2007 to 04/2012	Japan	Single-center Retrospective	IMA and LA	Coils	6.0	10	131	141	7	IMA: 100% (N.S.) and LAs: 79.8% (N.S.)	N.S.	E = 0 C = 2
Alerci et al. (45)	03/1999 to 12/2009	Switzerland	Single-center Retrospective	IMA and LA	Coils	60.5	56	64	120	8	96% (119/124)	N.S.	N.S.
Bonvini et al. (46)	03/1999 to 12/2001	Switzerland	Single-center Retrospective	IMA and LA	Coils	17.0	22	N.S.	22		IMA: 100% (14/14) and LAs: 65% (24/37)	N.S.	1
Sheehan et al. (27)	06/2001 to 06/2005	United States	Single-center Retrospective	IMA and LA	Coils	15.0	55	N.S.	55	6	44% (14/32)	N.S.	1
Branzan et al. (39)	09/2014 to 09/2019	Canada	Single-center Retrospective	IMA and LA	Coils	23.0	139	N.S.	105		N.S.	6	7
Rokosh et al. (43)	01/2009 to 11/2020	United States	Multi-center Retrospective	IMA and LA	N.S.	E = 15.0 C = 14.6	272	14,788	15,060		N.S.	E = 17 C = 621	E = 10 C = 960
Vaillant et al. (37)	11/2007 to 06/2016	France	Single-center Retrospective	IMA	Coils	E = 21.4 C = 57.2	33	45	78	9	100% (43/43)	E = 5 C = 14	E = 1 C = 15
Samura et al. (38)	04/2014 to 03/2018	Japan	Single-center Retrospective	IMA	AVP	E = 22.5 C = 22.4	46	51	97		88.7% (47/53)	E = 0 C = 0	E = 1 C = 9
Fukuda et al. (47)	07/2007 to 04/2014	Japan	Single-center Retrospective	IMA	Coils	E = 15.4 C = 37.5	143	189	332	7	N.S.	E = 1 C = 12	
Müller-Wille et al. (33)	07/2011 to 06/2013	Germany	Single-center Retrospective	IMA	AVP	E = 6.0 C = 26.0	31	43	74	6	100% (29/29)	N.S.	E = 0 C = 8
Ward et al. (31)	08/2002 to 05/2010	United States	Single-center Retrospective	IMA	Coils	E = 32.4 C = 21.2	108	158	266	6	100% (108/108)	E = 1 C = 12	E = 13 C = 21
Nevala et al. (28)	01/2000 to 10/2006	Finland	Multi-center Retrospective	IMA	Coils	E = 39.6 C = 40.8	40	39	79	7	84.8% (67/79)	E = 1 C = 11	N.S.
Axelrod et al. (26)	N.S.	United States	Single-center Retrospective	IMA	Coils	E = 6.0 C = 6.0	18	54	72	7	94% (30/32)	E = 0 C = 4	N.S.
Muthu et al. (48)	02/1999 to 04/2006	New Zealand	Single-center Retrospective	SAC	Thrombin	E = 12.0 C = 36.0	65	67	132		42% (29/69)	E = 2 C = 10	N.S.
Ronsivalle et al. (49)	01/2005 to 02/2008	Poland	Single-center Retrospective	SAC	Fibrin glue and Coils	E = 18.5 C = 20.0	18	20	38	9	100% (18/18)	E = 1 C = 2	N.S.
Pilon et al. (19)	09/1999 to 12/2008	Italy	Single-center Retrospective	SAC	Fibrin glue and Coils	E = 26.0 C = 72.0	180	224	404	9	N.S.	E = 14 C = 21	N.S.
Piazza et al. (30)	01/2008 to 12/2009	United States	Single-center Retrospective	SAC	Fibrin glue and Coils	E = 13.2 C = 37.2	79	83	162	9	100% (79/79)	E = 1 C = 5	E = 8 C = 21
Piazza et al. (36)	01/2012 to 12/2014	United States	Single-center Retrospective	SAC	Fibrin glue and Coils	E = 16.4 C = 15.9	50	55	105	9	100% (55/55)	E = 1 C = 6	N.S.
Mascoli et al. (34)	2008 to 2013	Italy	Single-center Retrospective	SAC	Coils	12.0	25	44	69	9	N.S.	E = 0 C = 0	E = 0 C = 5
Natrella et al. (17)	01/2011 to 04/2014	Italy	Single-center Retrospective	SAC	Coils	12.0	36	36	72	8	100% (36/36)	N.S.	N.S.
Dosluoglu et al. (18)	09/2007 to 10/2015	United States	Single-center Retrospective	SAC	Coils	44.0	16	31	47	9	93.7% (15/16)	E = 1 C = 9	E = 1 C = 14
Fabre et al. (40)	06/2014 to 02/2017	France	Multi-center Randomized Controlled Trial	SAC	Coils	24.0	29	32	61	9	100% (29/29)	E = 1 C = 7	E = 0 C = 0
Mascoli et al. (41)	01/2012 to 03/2015	Italy	Single-center Prospectively	SAC	Coils	12.0	61	265	326	9	N.S.	N.S.	N.S.
Zanchetta et al. (50)	06/2003 to 12/2005	Italy	Single-center Retrospective	SAC	Fibrin glue	14.4	84	N.S.	84		99% (83/84)	4	1
Nakai et al. (35)	12/2013 to 04/2015	Japan	Single-center Retrospective	SAC	Coils	12.1	24	N.S.	24		N.S.	N.S.	0
Mathlouthi et al. (42)	2015 to 2019	United States	Single-center Retrospective	SAC	Thrombin, morcellated gelfoam and non-heparinized saline	14.0	44	N.S.	44		100% (44/44)	3	0

## Results

### Primary and secondary outcomes

#### T2EL

**FIGURE 1:**
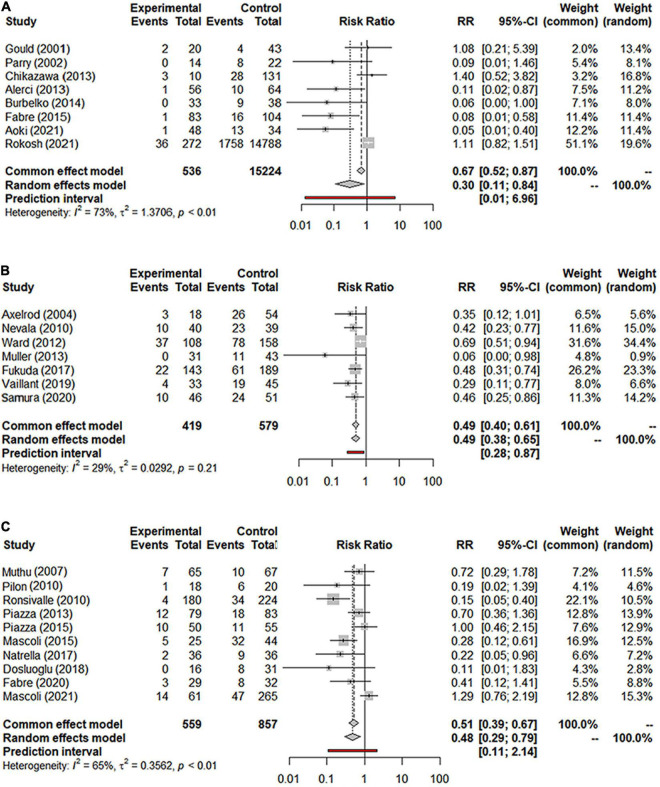
**(A)** Forest graph of T2EL in NS-ASSB group (RR 0.30 95% Cl 0.11–0.84); **(B)** forest graph of T2EL in IMA-ASSB group (RR 0.49 95% Cl 0.38–0.65); **(C)** forest graph of T2EL in ASCE group (RR 0.48 95% Cl 0.29–0.79). NS-ASSB, non-selective embolization of aneurysm sac side branches; IMA-ASSB, embolization of the inferior mesenteric artery; ASCE: aneurysm sac coil embolization.

**FIGURE 2:**
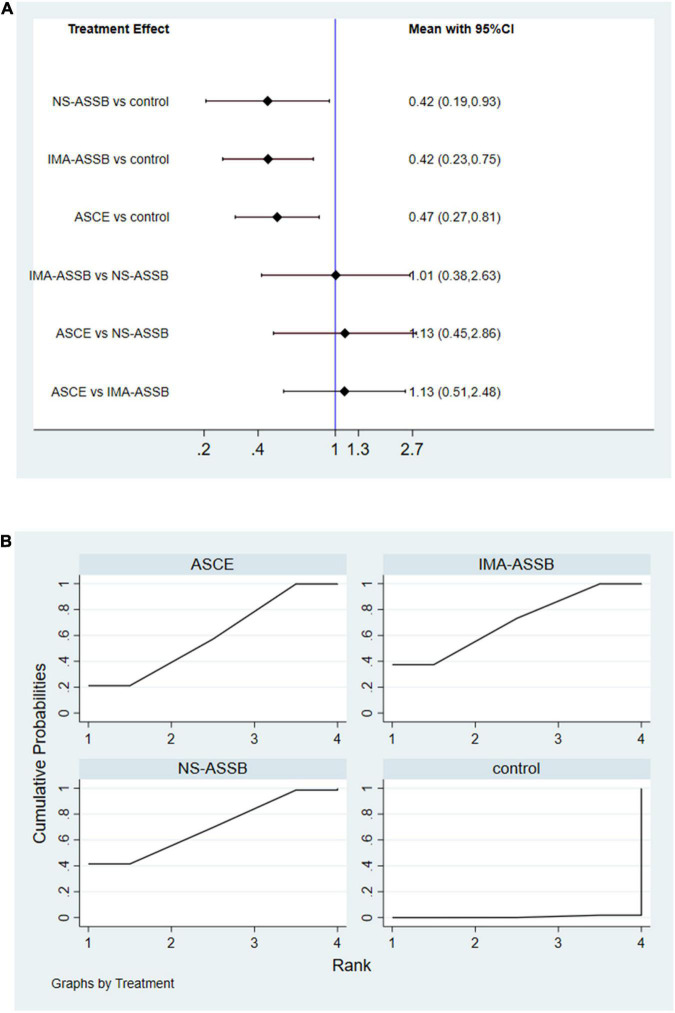
**(A)** Network meta-analysis forest graph of T2EL in NS-ASSB, IMA-ASSB and ASCE groups; **(B)** SUCRA curve of T2ELin NS-ASSB, IMA-ASSB and ASCE groups; NS-ASSB, non-selective embolization of aneurysm sac side branches; IMA-ASSB, embolization of the inferior mesenteric artery; ASCE, aneurysm sac coil embolization.

Overall, a total of 31 studies including 18,542 patients were analyzed (15,976 in the NS-ASSB group, 998 in the IMA-ASSB group, and 1,568 in the ASCE group). The prevalence of each embolization treatment was 0%–30% for NS-ASSB, 0%–34% for IMA-ASSB, and 0%–23.0% for ASCE. Patients who received prophylactic embolization had a significantly lower risk of T2EL after EVAR than those who did not receive prophylactic embolization in all studies. In the analysis of 25 controlled studies, the relative risk (RR) of T2EL was 0.30 (95% CI 0.11–0.84) for NS-ASSB, 0.49 (95% CI 0.38–0.65) for IMA-ASSB, and 0.48 (95% CI 0.29–0.79) for ASCE. Single-arm studies were not included in the routine meta-analysis because they contained smaller amounts of data. Network meta-analysis including single-arm studies using a multiple regression model demonstrated that both ASCE and IMA-ASSB were inferior to NS-ASSB in preventing T2EL. The effect of IMA-ASSB in preventing T2EL was similar (RR 1.01, 95% CI 0.38–2.63) to that of NS-ASSB and ASCE (RR 0.88, 95% CI 0.40–1.96). The results suggested that the protective effects of NS-ASSB and IMA-ASSB were not significantly different. The surface under the cumulative ranking (SUCRA) curve analysis showed a similar result (Figures 1, 2).

#### Re-intervention

**FIGURE 3:**
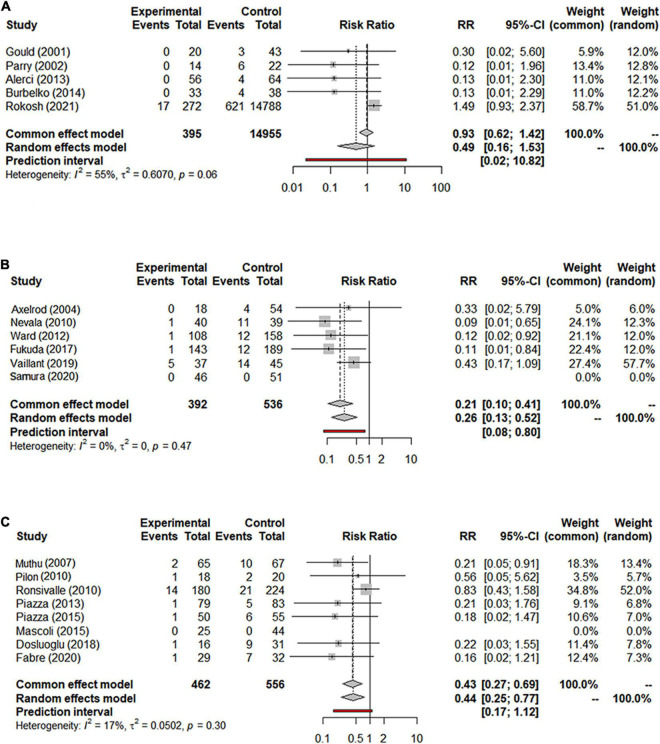
**(A)** Forest graph of re-intervention in NS-ASSB group (RR 0.49 95% Cl 0.16–1.53); **(B)** forest graph of re-intervention in IMA-ASSB group (RR 0.26 95% Cl 0.13–0.52); **(C)** forest graph of re-intervention in ASCE group (RR 0.44 95% Cl 0.25–0.77). NS-ASSB, non-selective embolization of aneurysm sac side branches; IMA-ASSB, embolization of the inferior mesenteric artery; ASCE, aneurysm sac coil embolization.

**FIGURE 4:**
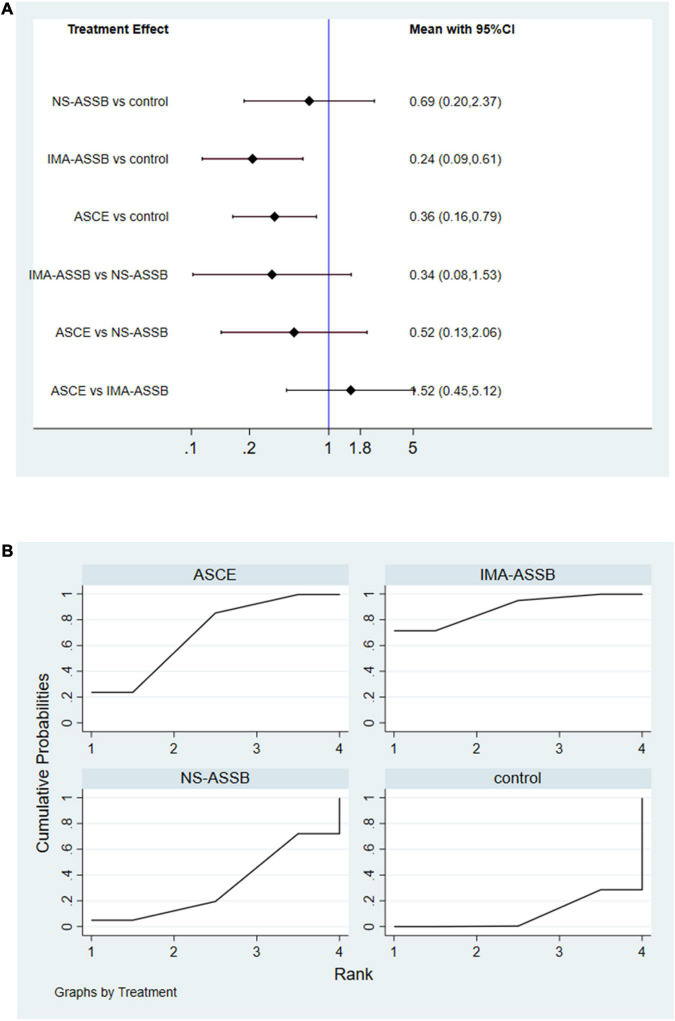
**(A)** Network meta-analysis forest graph of re-intervention in NS-ASSB, IMA-ASSB and ASCE groups; **(B)** SUCRA curve of re-intervention in NS-ASSB, IMA-ASSB and ASCE groups; NS-ASSB, non-selective embolization of aneurysm sac side branches; IMA-ASSB, embolization of the inferior mesenteric artery; ASCE, aneurysm sac coil embolization.

Data on the rate of re-intervention during follow-up were presented in 21 studies with a total of 17,405 patients (15,335 in the NS-ASSB group, 924 in the IMA-ASSB group, and 1,146 in the ASCE group). The rate of re-intervention ranged from 0% to 15.1% (0%–13.6% for NS-ASSB, 0%–15.1% for IMA-ASSB, and 0%–7.78% for ASCE). Prophylactic embolization resulted in a reduction in the incidence of re-intervention after EVAR. The RR of re-intervention in all controlled studies was 0.49 (95% CI 0.16–1.53) for NS-ASSB, 0.26 (95% CI 0.13–0.52) for IMA-ASSB, and 0.44 (95% CI 0.25–0.77) for ASCE. The single-arm studies were not analyzed because of a shortage of data. Network meta-analysis showed that IMA-ASSB was the best in preventing re-intervention after EVAR when all studies were incorporated in the analysis (RR 0.24, 95% CI 0.09–0.61) and was superior to NS-ASSB (RR 0.34, 95% CI 0.08–1.53) and ASCE (RR 0.66, 95% CI 0.19–2.22). The SUCRA curves were analyzed (Figures 3, 4).

#### Enlargement of the aneurysm sac

**FIGURE 5:**
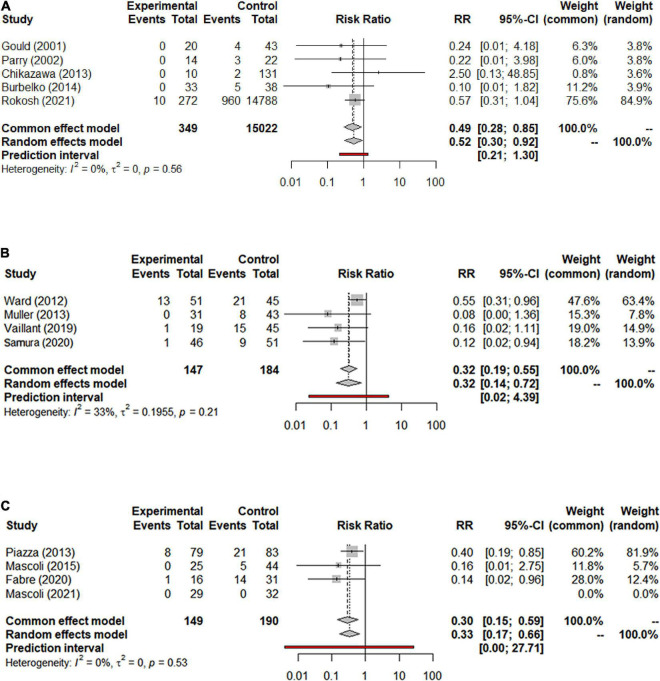
**(A)** Forest graph of enlargement of the aneurysm sac in NS-ASSB group (RR 0.52 95% Cl 0.30–0.92); **(B)** forest graph of enlargement of the aneurysm sac in IMA-ASSB group (RR 0.32 95% Cl 0.14–0.72); **(C)** forest graph of enlargement of the aneurysm sac in ASCE group (RR 0.33 95% Cl 0.17–0.66). NS-ASSB, non-selective embolization of aneurysm sac side branches; IMA-ASSB, embolization of the inferior mesenteric artery; ASCE, aneurysm sac coil embolization.

**FIGURE 6:**
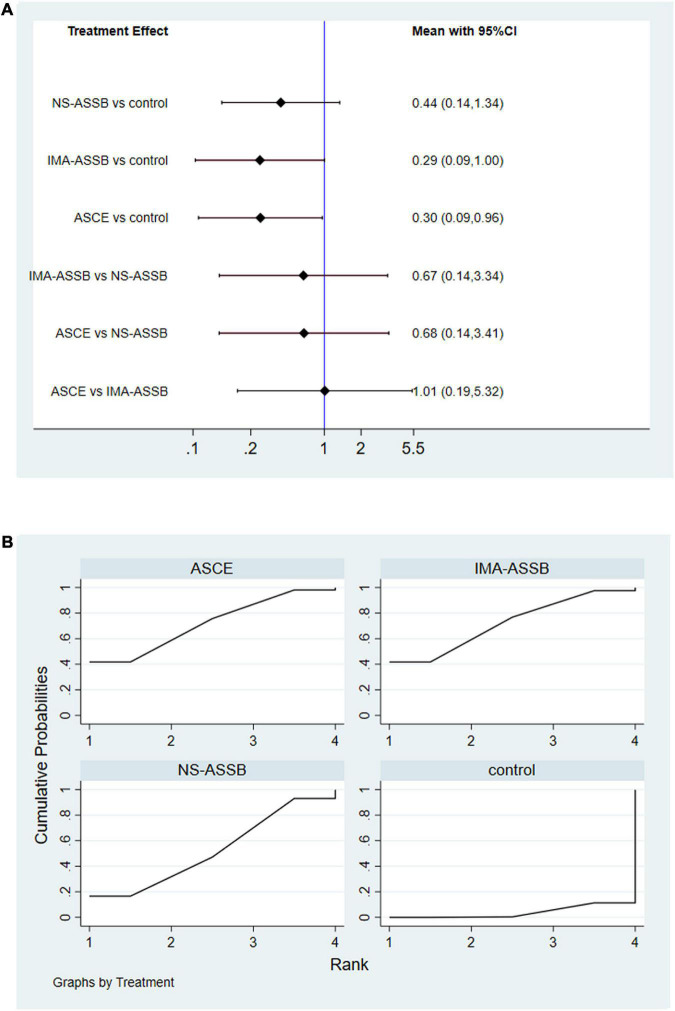
**(A)** Network meta-analysis forest graph of enlargement of the aneurysm sac in NS-ASSB, IMA-ASSB and ASCE groups; **(B)** SUCRA curve of enlargement of the aneurysm sac in NS-ASSB, IMA-ASSB and ASCE groups; NS-ASSB, non-selective embolization of aneurysm sac side branches; IMA-ASSB, embolization of the inferior mesenteric artery; ASCE, aneurysm sac coil embolization.

Data on the enlargement of the aneurysm sac were provided in 19 studies with a total of 16,559 patients (15,553 in the NS-ASSB group, 515 in the IMA-ASSB group, and 491 in the ASCE group). Aneurysm sac enlargement occurred in 0% to 12.0% of patients during follow-up (0%–3.7% for NS-ASSB, 0%–12.0% for IMA-ASSB, and 0%–10.1% for ASCE). The cumulative results showed that prophylactic embolization led to a significant decrease in the risk of aneurysm sac enlargement after EVAR. However, the results of individual studies showed the opposite conclusion. The RR of aneurysm sac enlargement in 13 controlled studies was 0.52 (95% CI 0.30–0.92) for NS-ASSB, 0.32 (95% CI 0.14–0.72) for IMA-ASSB, and 0.33 (95% CI 0.17–0.66) for ASCE. The single-arm studies were not analyzed because of a shortage of data. Network meta-analysis using a frequentist model proved that IMA-ASSB had the best performance in preventing enlargement of the aneurysm sac when all studies were incorporated in the analysis (RR 0.29, 95% CI 0.09–1.00), and was superior to NS-ASSB (RR 0.67, 95% CI 0.14–3.34) and similar to ASCE (RR 0.99, 95% CI 0.19–5.26). The same result was shown in the SUCRA curve analysis (Figures 5, 6).

## Discussion

The potential effects of T2EL after EVAR remain controversial. Therefore, there are still disputes regarding the management of T2EL and its influence on further outcomes. There is currently no consensus regarding the need for intensive treatment of T2EL. Evidence indicates that T2EL is not an isolated complication and is associated with the occurrence of other types of endoleaks. Despite the controversy over the past two decades around whether prophylactic embolization prevents T2EL, an increasing body of evidence suggests that such treatments may dramatically improve the outcome of EVAR. Studies have reported that prophylactic embolization has potential benefits in decreasing the incidence of T2EL, preventing enlargement of the aneurysm sac, and decreasing the rate of re-intervention (21, 51). The major limitation of these previous studies was a lack of a comparison of different interventions. However, it is difficult to make such comparisons in clinical practice because of the wide variations in the technical difficulty and cost of the interventions.

The clinical benefit of NS-ASSB has been discussed extensively since the first study was published in 2001 (15). The previous meta-analysis has given evidence of the safety and effectiveness of ASCE in preventing T2EL (21). Another meta-analysis shows a different rate of 19.9% vs. 41.4% in patients who accept IMA embolization or not (52). But there is still little information available on the comparison of the re-intervention rate and diameter change in different embolization strategies and no network meta-analysis is available until now. The present systematic review and network meta-analysis investigated the value of prophylactic embolization in preventing adverse outcomes after EVAR and compared different therapeutic regimens. We found that prophylactic embolization had a positive effect on the outcome of EVAR. Non-selective embolization of the IMA and LAs showed the best results in preventing T2EL, but embolization of the IMA alone might provide better benefits in suppressing the expansion of the aneurysm sac and reducing the re-intervention rate. Although all three methods lead to common effects of sac regression and free form re-intervention. The long-term effects of the rate of diameter reduction and the second operation seem to be better when embolization of IMA was carried out in isolation. LAs may play an important role in the outflow tract in sac regression after EVAR. The embolization of LAs may reduce outflow efficiency which decreases the rate of diameter reduction. As mentioned above, the effects of T2EL after EVAR still require verification; however, T2EL may eventually lead to aneurysm sac expansion (53). Aneurysm sac expansion is a predictor of late complications after EVAR (54, 55) and increases the risk of AAA rupture (56). The guidelines of the European Society for Vascular Surgery and prior studies recommend surgical intervention when the diameter of the aneurysm sac enlarges by 10–13 mm during follow-up using the same imaging modality (11, 53, 57). The presence of a T2EL and an increasing aneurysm sac size is likely to lead to a type I endoleak (57), which has a potential correlation with the risk of AAA rupture (58) and requires immediate treatment. There seems to be no consensus on the indications for re-intervention. A previous study assessed type I or III endoleaks, aneurysm sac expansion of more than 10 mm, and the existence of a collateralized IMA feeding vessel as indications for intervention (53). Our results indicated that prophylactic embolization of the IMA reduced the rate of aneurysm sac enlargement and improved the clinical outcomes of EVAR. These results suggest an underlying positive correlation between embolization of the LAs and expansion of the aneurysm sac diameter, which eventually increased the risk of re-intervention. Muthu et al. reported a high incidence of lumbar endoleaks after EVAR (48), which may result in the difference between the outcome of NS-ASSB and the stable optimal outcome of IMA-ASSB and ASCE demonstrated in the present study. More clinical data about the outcome of embolization of the LAs alone during EVAR is needed to confirm whether the hemodynamics are changed by this process or whether the hemodynamics are only changed by occlusion of the IMA. With regards to potential risks of ASSB and ASCE, Ward et (31) reported a 9.3% rate of complications among embolization patients, only one of them died because of multiorgan failure caused by colonic infarction after IMA embolization.

Although there were limited data available regarding the outcomes after prophylactic embolization during EVAR, even fewer data were available regarding the association of specific embolization treatments with aneurysm sac enlargement and re-intervention. Only one of the included studies was a randomized controlled trial, and most of the included studies had small sample sizes while one multicenter retrospective study had an extremely large sample size. Although the network meta-analysis was performed after the exclusion of the data from the large study by Rokosh et al. (43) showed similar results, bias may still exist. Eleven of the included studies were performed in the past 5 years, and most studies were performed over long time intervals that would bring bias caused by technological innovation. In addition, there were limited data on the incidences of aneurysm sac enlargement and re-intervention, and on complications after EVAR with versus without prophylactic embolization treatments. Furthermore, some patients did not receive embolization because they underwent emergency surgery, had unsuitable artery anatomy, or were physically unable to tolerate the procedure, which resulted in bias. Because all the calculated I^2^ values were less than 75%, publication bias was proved by funnel plots and Egger’s test (*p* = 0.0027 for the NS-ASSB group, *p* = 0.0252 for the IMA-ASSB group, and *p* = 0.0047 for the ASCE group). Besides, network meta-analysis has its advantages and drawbacks. Mvmeta package on Stata platform made based on multivariate regression analysis could obtain outcomes very close to Bayesian model. But only one dummy variable can be set in each operation. Moreover, heterogeneity and transitivity assumptions are still been challenged and stand in the way. To reduce the bias introduced by the performance of retrospective studies at single centers, more randomized controlled trials (preferably multicenter) are needed to verify the safety and efficacy of prophylactic embolization. Studies should be registered and carried out in accordance with the standard instructions for clinical trials. Blinded raters should perform CTA and assess the symptoms and disease progress at admission, every 6 or 12 months, and at discharge. Any CTA imaging data should be measured by independent reviewers using the same software tool. There should also be more precise definitions of aneurysm sac enlargement and indications for re-interventions.

## Conclusion

Prophylactic embolization during EVAR effectively prevents T2EL, suppresses the aneurysm sac expansion, and reduces the rate of re-intervention. Non-selective embolization of the IMA and LAs shows the best results in preventing T2EL. IMA embolization demonstrated certain benefits in achieving long-term aneurysm sac stability and lowering the risk of secondary surgery. Embolization of the LAs increases the operation time and medical expenses, but leads to potentially negative effects on the long-term outcome. We recommend conducting prophylactic embolization, especially IMA embolization alone or ASCE, to enhance the clinical benefits. More high-quality studies are needed to confirm the present findings.

## Data availability statement

The datasets presented in this study can be found in online repositories. The names of the repository/repositories and accession number(s) can be found in the article/Supplementary Material.

## Author contributions

YW and JY conceived the study, designed the method, contributed to the literature searches, provided statistical analysis, and wrote the manuscript. ZH investigated the literatures. GW excluded the documents. All authors interpreted the data, read the manuscript, and approved the final version.

## Conflict of interest

The authors declare that the research was conducted in the absence of any commercial or financial relationships that could be construed as a potential conflict of interest.

## Publisher’s note

All claims expressed in this article are solely those of the authors and do not necessarily represent those of their affiliated organizations, or those of the publisher, the editors and the reviewers. Any product that may be evaluated in this article, or claim that may be made by its manufacturer, is not guaranteed or endorsed by the publisher.

## References

[1] Greenhalgh RM Brown LC Kwong GP Powell JT Thompson SG EVAR trial participants. Comparison of endovascular aneurysm repair with open repair in patients with abdominal aortic aneurysm (EVAR trial 1), 30-day operative mortality results: randomised controlled trial. *Lancet.* (2004) 364:843–8. 15351191 10.1016/S0140-6736(04)16979-1

[2] Patel R Sweeting MJ Powell JT Greenhalgh RM EVAR trial investigators. Endovascular versus open repair of abdominal aortic aneurysm in 15-years’ follow-up of the UK endovascular aneurysm repair trial 1 (EVAR trial 1): a randomised controlled trial. *Lancet.* (2016) 388:2366–74. 10.1016/S0140-6736(16)31135-7 27743617

[3] De Bruin JL Baas AF Buth J Prinssen M Verhoeven EL Cuypers PW Long-term outcome of open or endovascular repair of abdominal aortic aneurysm. *N Engl J Med.* (2010) 362:1881–9. 20484396 10.1056/NEJMoa0909499

[4] Dijkstra ML Zeebregts CJ Verhagen HJM Teijink JAW Power AH Bockler D Incidence, natural course, and outcome of type II endoleaks in infrarenal endovascular aneurysm repair based on the ENGAGE registry data. *J Vasc Surg.* (2020) 71:780–9. 10.1016/j.jvs.2019.04.486 31443976

[5] Sidloff DA Gokani V Stather PW Choke E Bown MJ Sayers RD. Type II endoleak: conservative management is a safe strategy. *Eur J Vasc Endovasc Surg.* (2014) 48:391–9. 10.1016/j.ejvs.2014.06.035 25042332

[6] Rehman ZU. Endoleaks: current concepts and treatments - A narrative review. *J Pak Med Assoc.* (2021) 71:2224–9. 10.47391/JPMA.03-345 34580519

[7] Mulay S Geraedts ACM Koelemay MJW Balm R ODYSSEUS study group. Type 2 endoleak with or without intervention and survival after endovascular aneurysm repair. *Eur J Vasc Endovasc Surg.* (2021) 61:779–86. 10.1016/j.ejvs.2021.01.017 33632609

[8] Jones JE Atkins MD Brewster DC Chung TK Kwolek CJ LaMuraglia GM Persistent type 2 endoleak after endovascular repair of abdominal aortic aneurysm is associated with adverse late outcomes. *J Vasc Surg.* (2007) 46:1–8. 10.1016/j.jvs.2007.02.073 17543489

[9] Rayt HS Sandford RM Salem M Bown MJ London NJ Sayers RD. Conservative management of type 2 endoleaks is not associated with increased risk of aneurysm rupture. *Eur J Vasc Endovasc Surg.* (2009) 38:718–23. 10.1016/j.ejvs.2009.08.006 19767222

[10] El Batti S Cochennec F Roudot-Thoraval F Becquemin JP. Type II endoleaks after endovascular repair of abdominal aortic aneurysm are not always a benign condition. *J Vasc Surg.* (2013) 57:1291–7. 10.1016/j.jvs.2012.10.118 23465173

[11] Wanhainen A Verzini F Van Herzeele I. Editor’s Choice - European Society for Vascular Surgery (ESVS) 2019 clinical practice guidelines on the management of abdominal aorto-iliac artery aneurysms. *Eur J Vasc Endovasc Surg.* (2019) 57:8–93. Erratum in: Eur J Vasc Endovasc Surg. 2020;59(3):494. 10.1016/j.ejvs.2018.09.020 30528142

[12] Schlösser FJ Gusberg RJ Dardik A Lin PH Verhagen HJ Moll FL Aneurysm rupture after EVAR: can the ultimate failure be predicted? *Eur J Vasc Endovasc Surg.* (2009) 37:15–22. 19008129 10.1016/j.ejvs.2008.10.011

[13] Sidloff DA Stather PW Choke E Bown MJ Sayers RD. Type II endoleak after endovascular aneurysm repair. *Br J Surg.* (2013) 100:1262–70. 23939840 10.1002/bjs.9181

[14] Patel R Powell JT Sweeting MJ Epstein DM Barrett JK Greenhalgh RM. The UK EndoVascular Aneurysm Repair (EVAR) randomised controlled trials: long-term follow-up and cost-effectiveness analysis. *Health Technol Assess.* (2018) 22:1–132. 10.3310/hta22050 PMC5817412 29384470

[15] Gould DA McWilliams R Edwards RD Martin J White D Joekes E Aortic side branch embolization before endovascular aneurysm repair: incidence of type II endoleak. *J Vasc Interv Radiol.* (2001) 12:337–41. 11287511 10.1016/s1051-0443(07)61913-7

[16] Zhang H Yang Y Kou L Sun H Chen Z. Effectiveness of collateral arteries embolization before endovascular aneurysm repair to prevent type II endoleaks: a systematic review and meta-analysis. *Vascular.* (2021):17085381211032764. [Online ahead of print], 10.1177/17085381211032764 34266336

[17] Natrella M Rapellino A Navarretta F Iob G Cristoferi M Castagnola M Embo-EVAR: a technique to prevent Type II endoleak? A single-center experience. *Ann Vasc Surg.* (2017) 44:119–27. 10.1016/j.avsg.2017.01.028 28479464

[18] Dosluoglu HH Rivero M Khan SZ Cherr GS Harris LM Dryjski ML. Pre-emptive nonselective perigraft aortic sac embolization with coils to prevent type II endoleak after endovascular aneurysm repair. *J Vasc Surg.* (2019) 69:1736–46. 10.1016/j.jvs.2018.10.054 30591300

[19] Pilon F Tosato F Danieli D Campanile F Zaramella M Milite D. Intrasac fibrin glue injection after platinum coils placement: the efficacy of a simple intraoperative procedure in preventing type II endoleak after endovascular aneurysm repair. *Interact Cardiovasc Thorac Surg.* (2010) 11:78–82. 10.1510/icvts.2009.231167 20378698

[20] Fabre D Fadel E Brenot P Hamdi S Gomez Caro A Mussot S Type II endoleak prevention with coil embolization during endovascular aneurysm repair in high-risk patients. *J Vasc Surg.* (2015) 62:1–7. 25937609 10.1016/j.jvs.2015.02.030

[21] Li Q Hou P. Sac embolization and side branch embolization for preventing Type II endoleaks after endovascular aneurysm repair: a meta-analysis. *J Endovasc Ther.* (2020) 27:109–16. 31566053 10.1177/1526602819878411

[22] Liberati A Altman DG Tetzlaff J Mulrow C Gøtzsche PC Ioannidis JP The PRISMA statement for reporting systematic reviews and meta-analyses of studies that evaluate health care interventions: explanation and elaboration. *J Clin Epidemiol.* (2009) 62:e1–34. 19631507 10.1016/j.jclinepi.2009.06.006

[23] Wells G Shea B O’Connell D Peterson J Welch V Losos M *The Newcastle-Ottawa Scale (NOS) for Assessing The Quality Of Nonrandomised Studies In Meta-Analyses.* Ottawa: Ottawa Hospital Research Institute (2021).

[24] RStudio Team. *RStudio: Integrated Development Environment for R.* Boston, MA: RStudio, PBC (2021).

[25] Parry DJ Kessel DO Robertson I Denton L Patel JV Berridge DC Type II endoleaks: predictable, preventable, and sometimes treatable? *J Vasc Surg.* (2002) 36:105–10. 10.1067/mva.2002.125023 12096266

[26] Axelrod DJ Lookstein RA Guller J Nowakowski FS Ellozy S Carroccio A Inferior mesenteric artery embolization before endovascular aneurysm repair: technique and initial results. *J Vasc Interv Radiol.* (2004) 15:1263–7. 10.1097/01.RVI.0000141342.42484.90 15525746

[27] Sheehan MK Hagino RT Canby E Wholey MH Postoak D Suri R Type 2 endoleaks after abdominal aortic aneurysm stent grafting with systematic mesenteric and lumbar coil embolization. *Ann Vasc Surg.* (2006) 20:458–63. 10.1007/s10016-006-9103-2 16799851

[28] Nevala T Biancari F Manninen H Matsi P Mäkinen K Ylönen K Inferior mesenteric artery embolization before endovascular repair of an abdominal aortic aneurysm: effect on type II endoleak and aneurysm shrinkage. *J Vasc Interv Radiol.* (2010) 21:181–5. 10.1016/j.jvir.2009.10.014 20022764

[29] Chikazawa G Yoshitaka H Hiraoka A Tanaka K Mouri N Tamura K Preoperative coil embolization to aortic branched vessels for prevention of aneurysmal sac enlargement following EVAR: early clinical result. *Ann Vasc Dis.* (2013) 6:175–9. 10.3400/avd.oa.12.00079 23825498 PMC3692987

[30] Piazza M Frigatti P Scrivere P Bonvini S Noventa F Ricotta JJ II Role of aneurysm sac embolization during endovascular aneurysm repair in the prevention of type II endoleak-related complications. *J Vasc Surg.* (2013) 57:934–41. 10.1016/j.jvs.2012.10.078 23384494

[31] Ward TJ Cohen S Fischman AM Kim E Nowakowski FS Ellozy SH Preoperative inferior mesenteric artery embolization before endovascular aneurysm repair: decreased incidence of type II endoleak and aneurysm sac enlargement with 24-month follow-up. *J Vasc Interv Radiol.* (2013) 24:49–55. 10.1016/j.jvir.2012.09.022 23273697

[32] Burbelko M Kalinowski M Heverhagen JT Piechowiak E Kiessling A Figiel J Prevention of type II endoleak using the AMPLATZER vascular plug before endovascular aneurysm repair. *Eur J Vasc Endovasc Surg.* (2014) 47:28–36. 10.1016/j.ejvs.2013.10.003 24183247

[33] Müller-Wille R Uller W Gössmann H Heiss P Wiggermann P Dollinger M Inferior mesenteric artery embolization before endovascular aortic aneurysm repair using amplatzer vascular plug type 4. *Cardiovasc Interv Radiol.* (2014) 37:928–34. 10.1007/s00270-013-0762-4 24170169

[34] Mascoli C Freyrie A Gargiulo M Gallitto E Pini R Faggioli G Selective Intra-procedural AAA sac embolization during EVAR reduces the rate of Type II endoleak. *Eur J Vasc Endovasc Surg.* (2016) 51:632–9. 10.1016/j.ejvs.2015.12.009 26860254

[35] Nakai M Ikoma A Sato M Sato H Nishimura Y Okamura Y. Prophylactic intraoperative embolization of abdominal aortic aneurysm sacs using N-Butyl cyanoacrylate/lipiodol/ethanol mixture with proximal neck aortic balloon occlusion during endovascular abdominal aortic repair. *J Vasc Interv Radiol.* (2016) 27:954–60. 10.1016/j.jvir.2016.03.037 27234482

[36] Piazza M Squizzato F Zavatta M Menegolo M Ricotta JJ II Lepidi S Outcomes of endovascular aneurysm repair with contemporary volume-dependent sac embolization in patients at risk for Type II endoleak. *J Vasc Surg.* (2016) 63:32–8. 10.1016/j.jvs.2015.08.049 26432285

[37] Vaillant M Barral PA Mancini J De Masi M Bal L Piquet P Preoperative inferior mesenteric artery embolization is a cost-effective technique that may reduce the rate of aneurysm sac diameter enlargement and reintervention after EVAR. *Ann Vasc Surg.* (2019) 60:85–94. 10.1016/j.avsg.2019.03.012 31200030

[38] Samura M Morikage N Otsuka R Mizoguchi T Takeuchi Y Nagase T Endovascular aneurysm repair with inferior mesenteric artery embolization for preventing Type II endoleak: a prospective randomized controlled trial. *Ann Surg.* (2020) 271:238–44. 10.1097/SLA.0000000000003299 30946077

[39] Branzan D Geisler A Steiner S Doss M Matschuck M Scheinert D Type II endoleak and aortic aneurysm sac shrinkage after preemptive embolization of aneurysm sac side branches. *J Vasc Surg.* (2021) 73:1973–9.e1. 10.1016/j.jvs.2020.11.032 33278537

[40] Fabre D Mougin J Mitilian D Cochennec F Garcia Alonso C Becquemin JP Prospective, randomised two centre trial of endovascular repair of abdominal aortic aneurysm with or without sac embolisation. *Eur J Vasc Endovasc Surg.* (2021) 61:201–9. 10.1016/j.ejvs.2020.11.028 33342658

[41] Mascoli C Faggioli G Gallitto E Pini R Fenelli C Cercenelli L Tailored Sac embolization during EVAR for preventing persistent Type II endoleak. *Ann Vasc Surg.* (2021) 76:293–301. 10.1016/j.avsg.2021.01.118 33823259

[42] Mathlouthi A Guajardo I Al-Nouri O Malas M Barleben A. Prophylactic aneurysm embolization during EVAR is safe, improves sac regression and decreases the incidence of Type II endoleak. *Ann Vasc Surg.* (2021) 74:36–41. 10.1016/j.avsg.2020.12.060 33549781

[43] Rokosh RS Chang H Butler JR Rockman CB Patel VI Milner R Prophylactic sac outflow vessel embolization is associated with improved sac regression in patients undergoing endovascular aortic aneurysm repair. *J Vasc Surg.* (2021) 76:113–21.e8. 10.1016/j.jvs.2021.11.070 34923066

[44] Aoki A Maruta K Omoto T Masuda T. Midterm outcomes of endovascular abdominal aortic aneurysm repair with prevention of Type 2 endoleak by intraoperative aortic side branch coil embolization. *Ann Vasc Surg.* (2022) 78:180–9. 10.1016/j.avsg.2021.06.037 34537351

[45] Alerci M Giamboni A Wyttenbach R Porretta AP Antonucci F Bogen M Endovascular abdominal aneurysm repair and impact of systematic preoperative embolization of collateral arteries: endoleak analysis and long-term follow-up. *J Endovasc Ther.* (2013) 20:663–71. 10.1583/12-4188MR.1 24093319

[46] Bonvini R Alerci M Antonucci F Tutta P Wyttenbach R Bogen M Preoperative embolization of collateral side branches: a valid means to reduce type II endoleaks after endovascular AAA repair. *J Endovasc Ther.* (2003) 10:227–32. 10.1177/152660280301000210 12877603

[47] Fukuda T Matsuda H Tanaka H Sanda Y Morita Y Seike Y. Selective Inferior mesenteric artery embolization during endovascular abdominal aortic aneurysm repair to prevent Type II endoleak. *Kobe J Med Sci.* (2018) 63:E130–5. 30617246 PMC6345414

[48] Muthu C Maani J Plank LD Holden A Hill A. Strategies to reduce the rate of type II endoleaks: routine intraoperative embolization of the inferior mesenteric artery and thrombin injection into the aneurysm sac. *J Endovasc Ther.* (2007) 14:661–8. 10.1177/152660280701400509 17924731

[49] Ronsivalle S Faresin F Franz F Rettore C Zanchetta M Olivieri A. Aneurysm sac “thrombization” and stabilization in EVAR: a technique to reduce the risk of type II endoleak. *J Endovasc Ther.* (2010) 17:517–24. 10.1583/09-3004.1 20681769

[50] Zanchetta M Faresin F Pedon L Ronsivalle S. Intraoperative intrasac thrombin injection to prevent type II endoleak after endovascular abdominal aortic aneurysm repair. *J Endovasc Ther.* (2007) 14:176–83. 10.1177/152660280701400209 17484533

[51] Yu HYH Lindström D Wanhainen A Tegler G Hassan B Mani K. Systematic review and meta-analysis of prophylactic aortic side branch embolization to prevent type II endoleaks. *J Vasc Surg.* (2020) 72:1783–92.e1. 10.1016/j.jvs.2020.05.020 32442608

[52] Biancari F Mäkelä J Juvonen T Venermo M. Is inferior mesenteric artery embolization indicated prior to endovascular repair of abdominal aortic aneurysm? *Eur J Vasc Endovascular Surg.* (2015) 50:671–4. 10.1016/j.ejvs.2015.06.116 26319477

[53] Ajalat M Williams R Wilson SE. The natural history of type 2 endoleaks after endovascular aneurysm repair justifies conservative management. *Vascular.* (2018) 26:524–30. 10.1177/1708538118766103 29566590

[54] Bastos Gonçalves F Baderkhan H Verhagen HJ Wanhainen A Björck M Stolker RJ Early sac shrinkage predicts a low risk of late complications after endovascular aortic aneurysm repair. *Br J Surg.* (2014) 101:802–10. 10.1002/bjs.9516 24752772 PMC4164270

[55] Schanzer A Greenberg RK Hevelone N Robinson WP Eslami MH Goldberg RJ Predictors of abdominal aortic aneurysm sac enlargement after endovascular repair. *Circulation.* (2011) 123:2848–55. Erratum in: Circulation. 2012;125(2):e266. 10.1161/CIRCULATIONAHA.110.014902 21478500

[56] Chaikof E Dalman R Eskandari M Jackson B Lee W Mansour M The society for vascular surgery practice guidelines on the care of patients with an abdominal aortic aneurysm. *J Vasc Surg.* (2018) 67:2–77. 10.1016/j.jvs.2017.10.044 29268916

[57] Eden CL Long GW Major M Studzinski D Brown OW. Type II endoleak with an enlarging aortic sac after endovascular aneurysm repair predisposes to the development of a type IA endoleak. *J Vasc Surg.* (2020) 72:1354–9. 10.1016/j.jvs.2020.01.038 32417014

[58] van Marrewijk C Buth J Harris PL Norgren L Nevelsteen A Wyatt MG. Significance of endoleaks after endovascular repair of abdominal aortic aneurysms: the EUrosTAR experience. *J Vasc Surg.* (2002) 35:461–73. 10.1067/mva.2002.118823 11877693

